# Supplementary material for: Antiviral Effects of 5-Aminolevulinic Acid Phosphate against Classical Swine Fever Virus: In Vitro and In Vivo Evaluation
Source: Pathogens. 2022 Jan 27;11(2):164. doi: 10.3390/pathogens11020164 (PMC8877771; doi:10.3390/pathogens11020164)
Supplement: Supplementary file 1 [file pathogens-11-00164-s001.zip › Supplemental document20211231PM9 Jan22.pdf]

Supplemental document

Materials and Methods

Cytotoxicity assay

The cytotoxicity of each compound was measured by the MTT assay as described previously [1]. Briefly, after 2 days of incubation with the compounds, cells were incubated at 37°C for 2 hours with 5 mg/mL of 3-(4,5-di-methylthiazol-2-yl)-2,5-diphenyltetrazolium bromide (MTT: Dojindo laboratories, Kumamoto, Japan). Afterward, the solution was replaced with a cell lysis solution consisting of isopropanol (Nacalai tesque, Kyoto, Japan) with 10% TritonX-100 (FUJIFILM Wako Pure Chemical Corp., Osaka, Japan) and 0.3% hydrochloric acid (FUJIFILM Wako Pure Chemical Corp.). After overnight incubation at room temperature, the absorbance at 560 nm and 690 nm was measured by POWERSCAN4 (DS Pharma Biomedical, Osaka, Japan), and cell viability was calculated from the survival rate of the compound-treated cells compared to untreated cells. To determine the cytotoxic concentration of the stock solutions of each compound, stock solutions were prepared for serial dilutions as the concentration of 10 mM 5-ALA, 2.5 mM SFC, and 10 mM PPIX, respectively. Cells were suspended in the concentration of 5×10^5 cells/mL with 10% HS EMEM. Then, 100 μL of cell suspension was mixed with 50 μL of the compound in a 96-well plate and incubated at 37°C for 2 days in the presence of 5% CO_2.

Estimation of G4 structure in the viral genome of vALD-A76

The putative locus of the G4 structure in vALD-A76 was predicted by quadruplex forming G-rich sequences (QGRS) Mapper, a web-based server [2]. According to the QGRS method, putative G4s were identified by the motif GxNy1GxNy2GxNy3Gx, where x represents the number of guanine tetrads and y was the length of gaps. This analysis was conducted under the following restrictions: the maximum length is 30 bases, minimum G-tetrads equal to 2, loop size is from 0 to 36 and overlaps are not included. In addition, G-scores, scoring systems that evaluate the stability of G4, were calculated by QGRS Mapper.

Half-maximal effective concentration (EC_{50})

Antiviral effects of each compound were determined in the presence or absence of infectious virus, and EC_{50} was estimated based on the maximum and minimum value by using ImageJ (version 1.8.0, National Institutes of Health, MD, USA) [3]; the values of EC_{50} of each compound against CSFV vALD-A76 strain were measured using SK-L cells. First, 100 μL of cell suspension (5×10^4 cells) was mixed with the serially diluted compounds in a 96-well plate at room temperature for 1 hour. Then, 50 μL of the virus containing 5 $TCID_{50}$ was inoculated to the mixture. After 2 days incubation at 37°C in the presence of 5% CO_2, the plate was immunostained to calculate the virus titer under the supplementation of the compounds.

Serum neutralization test

Sera of the pigs were collected before the experiments and on the day of euthanasia. Serum neutralization tests were conducted by following the protocol described previously [4,5]. Briefly, an equal volume of each serum was inactivated in the presence of PBS with 0.3% of Tween 20 (Nacalai tesque) for 30 minutes at 56°C beforehand, and 100 $TCID_{50}$ of CSFV vALD-A76 was mixed and incubated at 37°C for 1 hour. The mixture was added to an SK-L cell suspension and incubated in 96-well plates at 37°C in the presence of 5% CO_2. Virus infection was confirmed by immunostaining on 72–96 hours post-inoculation. Neutralizing antibody titer was expressed as the reciprocal of the highest serum dilution which showed complete neutralization of the inoculated virus.

References

1. Mosmann, T. Rapid colorimetric assay for cellular growth and survival: Application to proliferation and cytotoxicity assays. *J. Immunol. Methods* **1983**, *65*, 55–63, doi:10.1016/0022-1759(83)90303-4.
2. Kikin, O.; D'Antonio, L.; Bagga, P.S. QGRS Mapper: A web-based server for predicting G-quadruplexes in nucleotide sequences. *Nucleic Acids Res.* **2006**, *34*, W676–W682, doi:10.1093/nar/gkl253.
3. Schneider, C.A.; Rasband, W.S.; Eliceiri, K.W. NIH Image to ImageJ: 25 years of image analysis. *Nat. Methods* **2012**, *9*, 671–675, doi:10.1038/nmeth.2089.
4. Sakoda, Y.; Wakamoto, H.; Tamura, T.; Nomura, T.; Naito, M.; Aoki, H.; Morita, H.; Kida, H.; Fukusho, A. Development and evaluation of indirect enzyme-linked immunosorbent assay for a screening test to detect antibodies against classical swine fever virus. *Jpn. J. Vet. Res.* **2012**, *60*, 85–94, doi:10.14943/jjvr.60.2-3.85.
5. Meyer, D.; Petrov, A.; Becher, P. Inactivation of classical swine fever virus in porcine serum samples intended for antibody detection. *Pathogens* **2019**, *8*, 286, doi:10.3390/pathogens8040286.
