# Supplementary material for: Antiviral Effects of 5-Aminolevulinic Acid Phosphate against Classical Swine Fever Virus: In Vitro and In Vivo Evaluation
Source: Pathogens. 2022 Jan 27;11(2):164. doi: 10.3390/pathogens11020164 (PMC8877771; doi:10.3390/pathogens11020164)
Supplement: Supplementary file 1 [file pathogens-11-00164-s001.zip › Supplemental fig_20211231AM9 Jan22.pptx]

## Slide 1
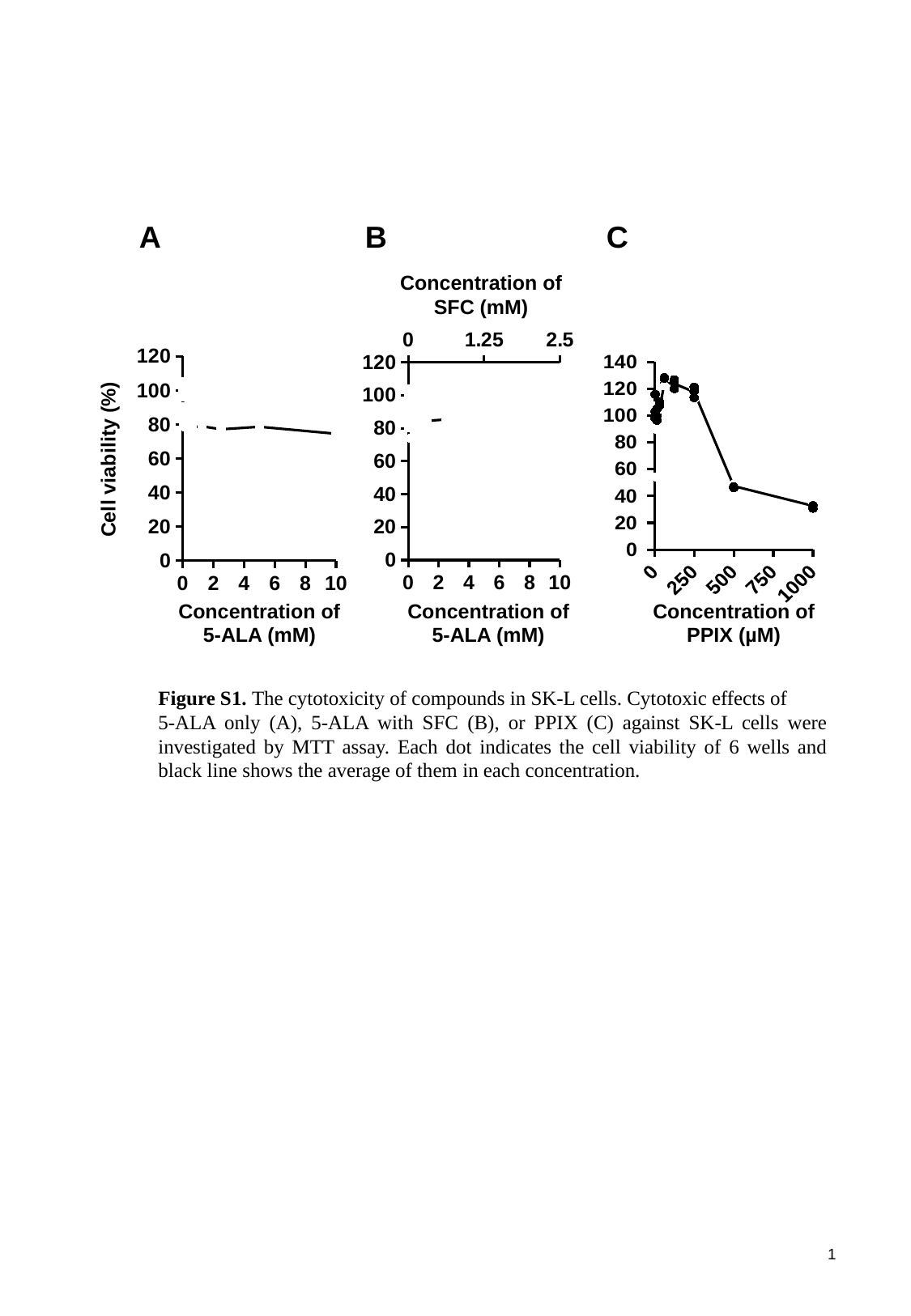

A
B
C
Concentration of
SFC (mM)
### Chart
| Category | | | | | | | | | |
|---|---|---|---|---|---|---|---|---|---|
### Chart
| Category | | | | | | | | | |
|---|---|---|---|---|---|---|---|---|---|
### Chart
| Category | | | | | | | |
|---|---|---|---|---|---|---|---|Cell viability (%)
Concentration of
5-ALA (mM)
Concentration of
5-ALA (mM)
Concentration of
PPIX (µM)
Figure S1. The cytotoxicity of compounds in SK-L cells. Cytotoxic effects of
5-ALA only (A), 5-ALA with SFC (B), or PPIX (C) against SK-L cells were investigated by MTT assay. Each dot indicates the cell viability of 6 wells and black line shows the average of them in each concentration.
1

## Slide 2
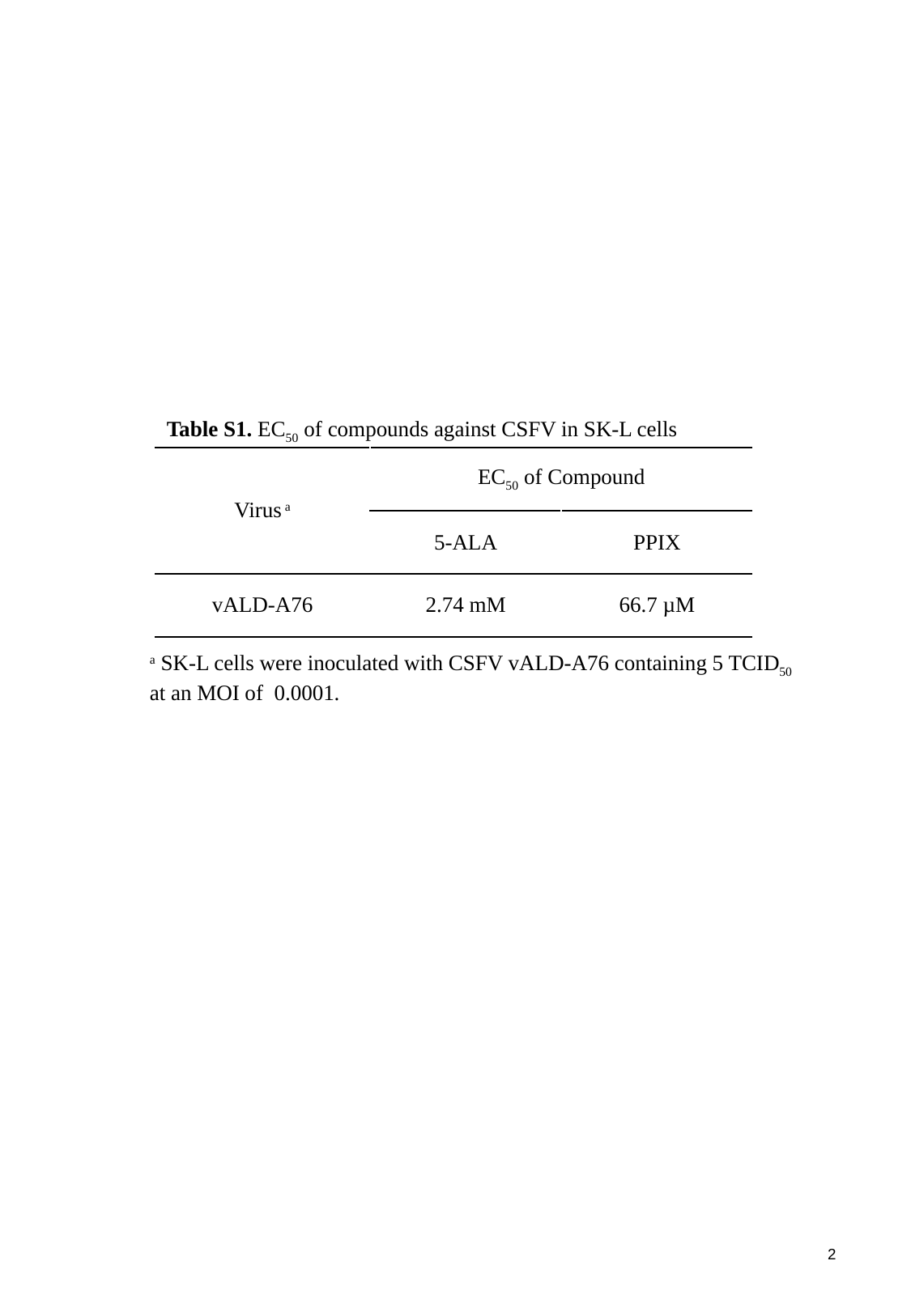

Table S1. EC50 of compounds against CSFV in SK-L cells
| Virus a | EC50 of Compound | (µM) |
| --- | --- | --- |
| | 5-ALA | PPIX |
| vALD-A76 | 2.74 mM | 66.7 µM |
a SK-L cells were inoculated with CSFV vALD-A76 containing 5 TCID50 at an MOI of 0.0001.
2

## Slide 3
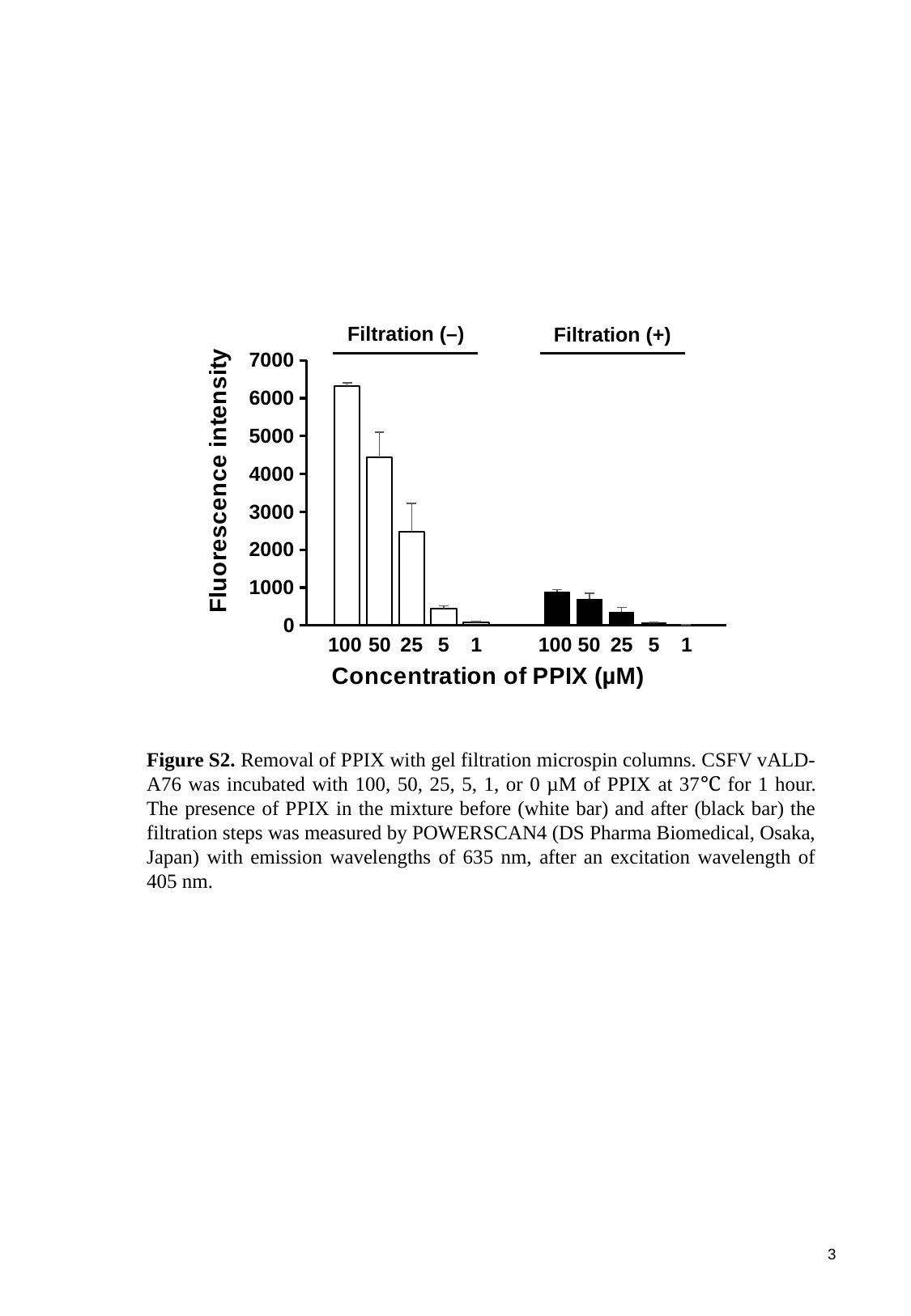

### Chart
| Category | 100 | 50 | 25 | 5 | 1 |
|---|---|---|---|---|---|
| before | 6312.666666666667 | 4438.5 | 2480.5 | 454.0 | 84.0 |
| after | 897.6666666666666 | 693.0 | 355.0 | 71.0 | 15.0 |Filtration (–)
Filtration (+)
5
25
25
100
50
5
1
100
50
1
Figure S2. Removal of PPIX with gel filtration microspin columns. CSFV vALD-A76 was incubated with 100, 50, 25, 5, 1, or 0 µM of PPIX at 37℃ for 1 hour. The presence of PPIX in the mixture before (white bar) and after (black bar) the filtration steps was measured by POWERSCAN4 (DS Pharma Biomedical, Osaka, Japan) with emission wavelengths of 635 nm, after an excitation wavelength of 405 nm.
3

## Slide 4
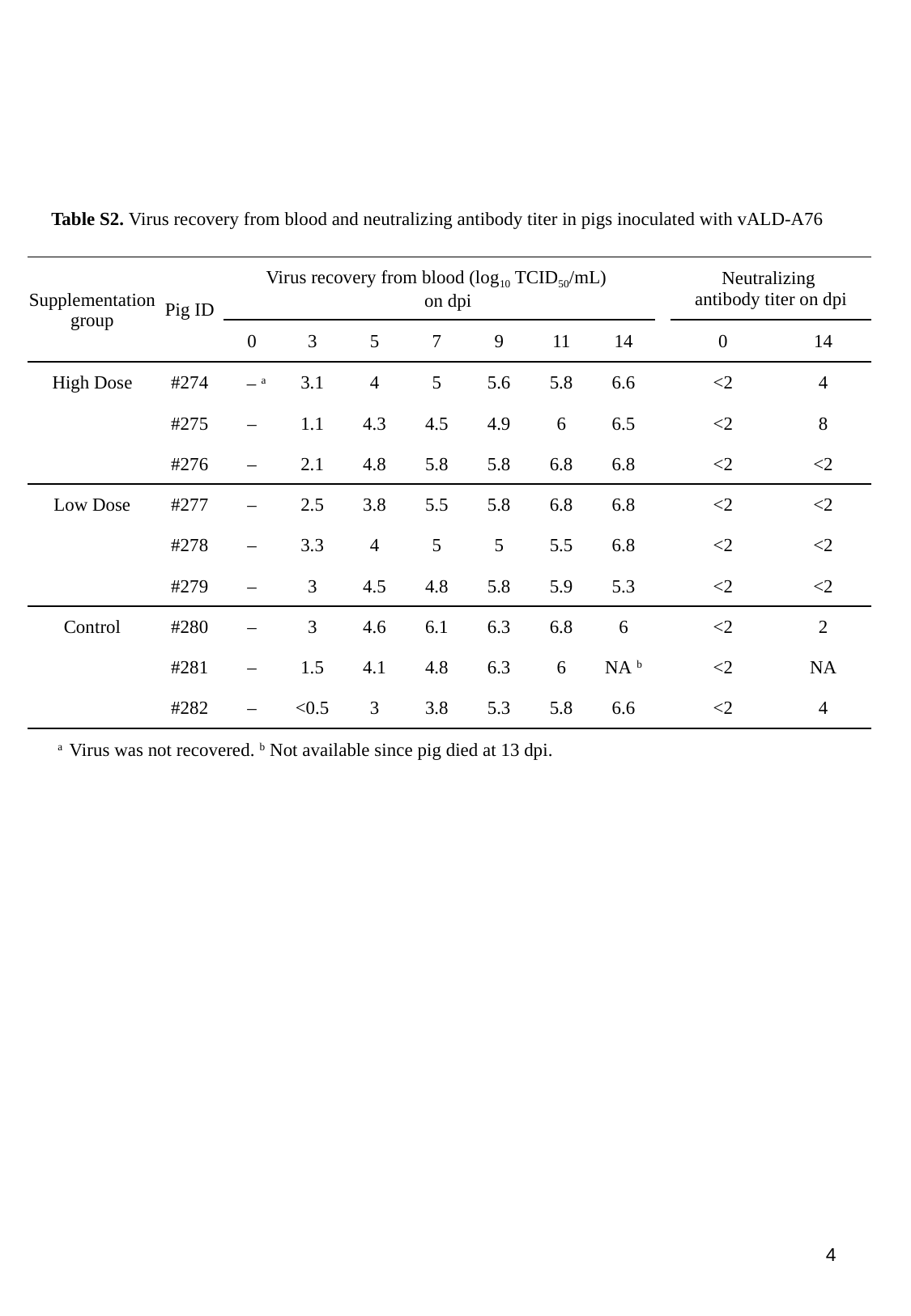

Table S2. Virus recovery from blood and neutralizing antibody titer in pigs inoculated with vALD-A76
| Supplementation group | Pig ID | Virus recovery from blood (log10 TCID50/mL) on dpi | | | | | | | | Neutralizing antibody titer on dpi | |
| --- | --- | --- | --- | --- | --- | --- | --- | --- | --- | --- | --- |
| | | 0 | 3 | 5 | 7 | 9 | 11 | 14 | | 0 | 14 |
| High Dose | #274 | – a | 3.1 | 4 | 5 | 5.6 | 5.8 | 6.6 | | <2 | 4 |
| | #275 | – | 1.1 | 4.3 | 4.5 | 4.9 | 6 | 6.5 | | <2 | 8 |
| | #276 | – | 2.1 | 4.8 | 5.8 | 5.8 | 6.8 | 6.8 | | <2 | <2 |
| Low Dose | #277 | – | 2.5 | 3.8 | 5.5 | 5.8 | 6.8 | 6.8 | | <2 | <2 |
| | #278 | – | 3.3 | 4 | 5 | 5 | 5.5 | 6.8 | | <2 | <2 |
| | #279 | – | 3 | 4.5 | 4.8 | 5.8 | 5.9 | 5.3 | | <2 | <2 |
| Control | #280 | – | 3 | 4.6 | 6.1 | 6.3 | 6.8 | 6 | | <2 | 2 |
| | #281 | – | 1.5 | 4.1 | 4.8 | 6.3 | 6 | NA b | | <2 | NA |
| | #282 | – | <0.5 | 3 | 3.8 | 5.3 | 5.8 | 6.6 | | <2 | 4 |
a Virus was not recovered. b Not available since pig died at 13 dpi.
4

## Slide 5
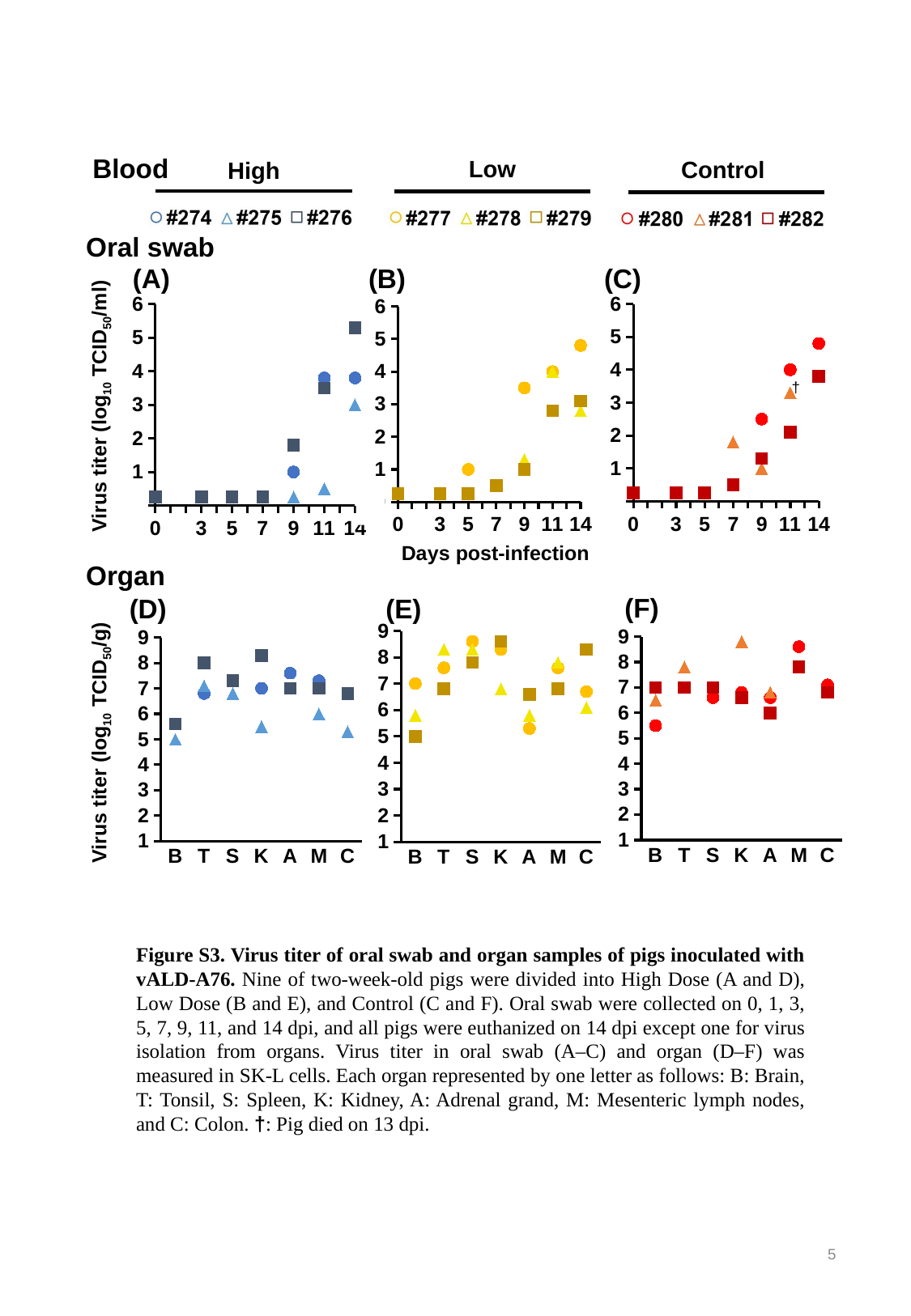

Blood
Low
Control
High
Oral swab
(A)
(C)
(B)
Virus titer (log10 TCID50/ml)
### Chart
| Category | #274 | #275 | #276 |
|---|---|---|---|
| 0 | 0.25 | 0.25 | 0.25 |
| | None | None | None |
| | None | None | None |
| 3 | 0.25 | 0.25 | 0.25 |
| | None | None | None |
| 5 | 0.25 | 0.25 | 0.25 |
| | None | None | None |
| 7 | 0.25 | 0.25 | 0.25 |
| | None | None | None |
| 9 | 1.0 | 0.25 | 1.8 |
| | None | None | None |
| 11 | 3.8 | 0.5 | 3.5 |
| | None | None | None |
| 14 | 3.8 | 3.0 | 5.3 |
### Chart
| Category | #280 | #281 | #282 |
|---|---|---|---|
| 0 | 0.25 | 0.25 | 0.25 |
| | None | None | None |
| | None | None | None |
| 3 | 0.25 | 0.25 | 0.25 |
| | None | None | None |
| 5 | 0.25 | 0.25 | 0.25 |
| | None | None | None |
| 7 | 0.5 | 1.8 | 0.5 |
| | None | None | None |
| 9 | 2.5 | 1.0 | 1.3 |
| | None | None | None |
| 11 | 4.0 | 3.3 | 2.1 |
| | None | None | None |
| 14 | 4.8 | None | 3.8 |
### Chart
| Category | #277 | #278 | #279 |
|---|---|---|---|
| 0 | 0.25 | 0.25 | 0.25 |
| | None | None | None |
| | None | None | None |
| 3 | 0.25 | 0.25 | 0.25 |
| | None | None | None |
| 5 | 1.0 | 0.25 | 0.25 |
| | None | None | None |
| 7 | 0.5 | 0.5 | 0.5 |
| | None | None | None |
| 9 | 3.5 | 1.3 | 1.0 |
| | None | None | None |
| 11 | 4.0 | 4.0 | 2.8 |
| | None | None | None |
| 14 | 4.8 | 2.8 | 3.1 |†
\\
Days post-infection
Organ
(F)
Virus titer (log10 TCID50/g)
(D)
(E)
### Chart
| Category | #277 | #278 | #279 |
|---|---|---|---|
| B | 7.0 | 5.8 | 5.0 |
| T | 7.6 | 8.3 | 6.8 |
| S | 8.6 | 8.3 | 7.8 |
| K | 8.3 | 6.8 | 8.6 |
| A | 5.3 | 5.8 | 6.6 |
| M | 7.6 | 7.8 | 6.8 |
| C | 6.7 | 6.1 | 8.3 |
### Chart
| Category | #280 | #281 | #282 |
|---|---|---|---|
| B | 5.5 | 6.5 | 7.0 |
| T | 7.0 | 7.8 | 7.0 |
| S | 6.6 | 7.0 | 7.0 |
| K | 6.8 | 8.8 | 6.6 |
| A | 6.6 | 6.8 | 6.0 |
| M | 8.6 | 7.8 | 7.8 |
| C | 7.1 | 6.8 | 6.8 |
### Chart
| Category | #274 | #275 | #276 |
|---|---|---|---|
| B | 5.6 | 5.0 | 5.6 |
| T | 6.8 | 7.1 | 8.0 |
| S | 7.3 | 6.8 | 7.3 |
| K | 7.0 | 5.5 | 8.3 |
| A | 7.6 | 7.0 | 7.0 |
| M | 7.3 | 6.0 | 7.0 |
| C | 6.8 | 5.3 | 6.8 |Figure S3. Virus titer of oral swab and organ samples of pigs inoculated with vALD-A76. Nine of two-week-old pigs were divided into High Dose (A and D), Low Dose (B and E), and Control (C and F). Oral swab were collected on 0, 1, 3, 5, 7, 9, 11, and 14 dpi, and all pigs were euthanized on 14 dpi except one for virus isolation from organs. Virus titer in oral swab (A–C) and organ (D–F) was measured in SK-L cells. Each organ represented by one letter as follows: B: Brain, T: Tonsil, S: Spleen, K: Kidney, A: Adrenal grand, M: Mesenteric lymph nodes, and C: Colon. †: Pig died on 13 dpi.
5

## Slide 6
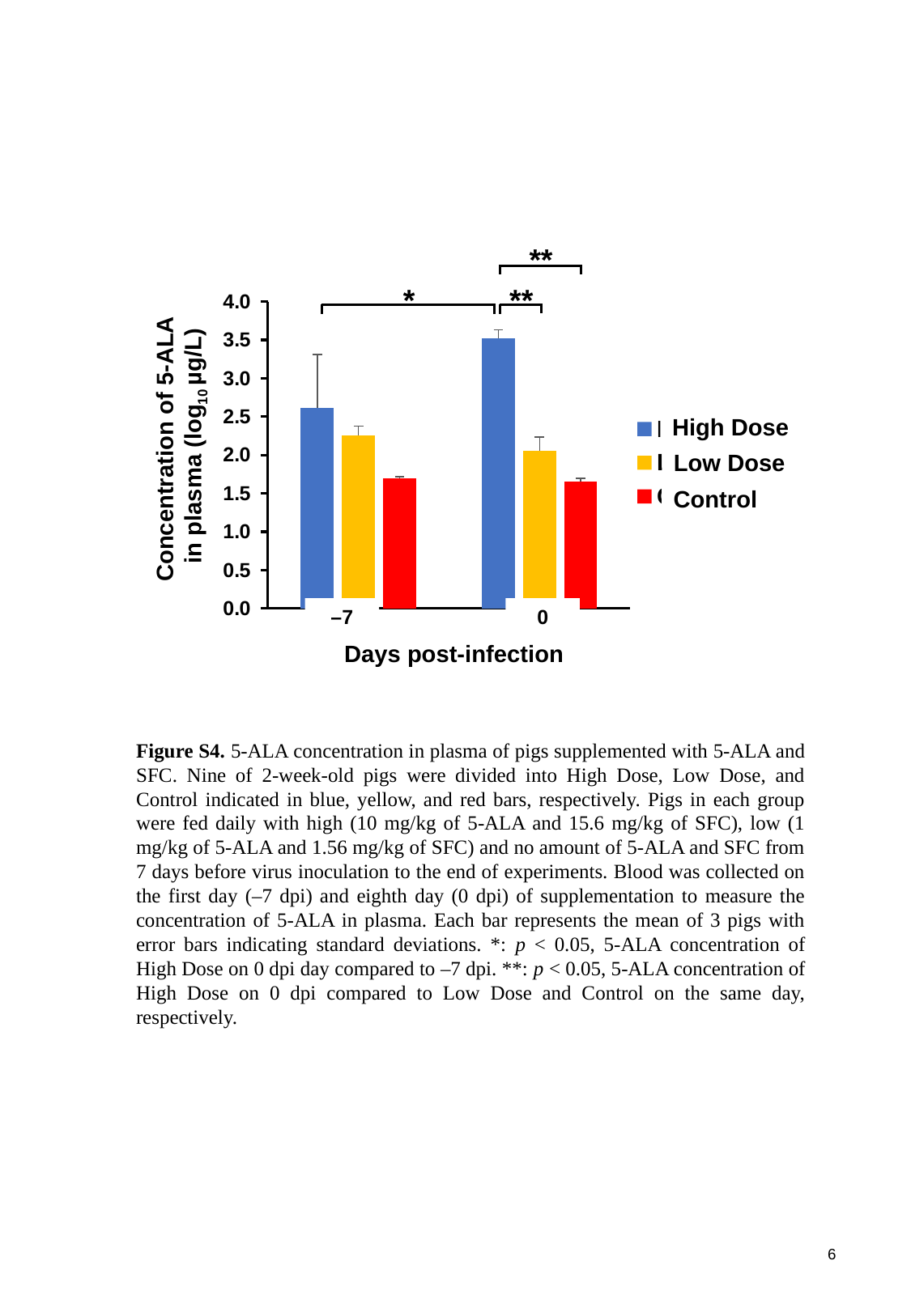

**
**
*
### Chart
| Category | High dose | Low dose | Control |
|---|---|---|---|
| -7d | 2.610323265161203 | 2.2559460136528835 | 1.6948533975518807 |
| 0d | 3.522569349513457 | 2.055868326649661 | 1.650109930977375 |High Dose
Concentration of 5-ALA
in plasma (log10 µg/L)
Low Dose
Control
0
–7
Days post-infection
Figure S4. 5-ALA concentration in plasma of pigs supplemented with 5-ALA and SFC. Nine of 2-week-old pigs were divided into High Dose, Low Dose, and Control indicated in blue, yellow, and red bars, respectively. Pigs in each group were fed daily with high (10 mg/kg of 5-ALA and 15.6 mg/kg of SFC), low (1 mg/kg of 5-ALA and 1.56 mg/kg of SFC) and no amount of 5-ALA and SFC from 7 days before virus inoculation to the end of experiments. Blood was collected on the first day (–7 dpi) and eighth day (0 dpi) of supplementation to measure the concentration of 5-ALA in plasma. Each bar represents the mean of 3 pigs with error bars indicating standard deviations. *: p < 0.05, 5-ALA concentration of High Dose on 0 dpi day compared to –7 dpi. **: p < 0.05, 5-ALA concentration of High Dose on 0 dpi compared to Low Dose and Control on the same day, respectively.
6

## Slide 7
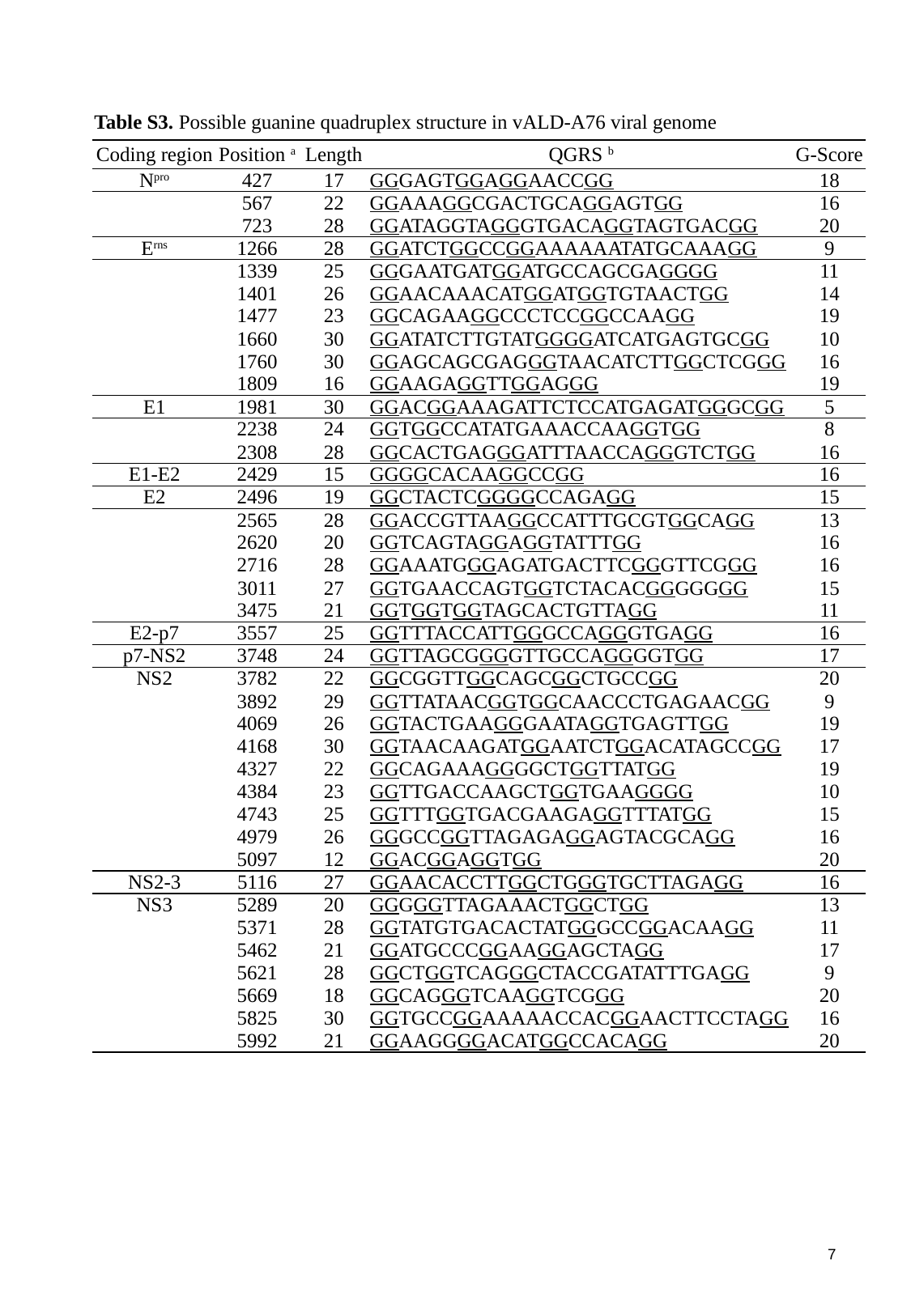

Table S3. Possible guanine quadruplex structure in vALD-A76 viral genome
| Coding region | Position a | Length | QGRS b | G-Score |
| --- | --- | --- | --- | --- |
| Npro | 427 | 17 | GGGAGTGGAGGAACCGG | 18 |
| | 567 | 22 | GGAAAGGCGACTGCAGGAGTGG | 16 |
| | 723 | 28 | GGATAGGTAGGGTGACAGGTAGTGACGG | 20 |
| Erns | 1266 | 28 | GGATCTGGCCGGAAAAAATATGCAAAGG | 9 |
| | 1339 | 25 | GGGAATGATGGATGCCAGCGAGGGG | 11 |
| | 1401 | 26 | GGAACAAACATGGATGGTGTAACTGG | 14 |
| | 1477 | 23 | GGCAGAAGGCCCTCCGGCCAAGG | 19 |
| | 1660 | 30 | GGATATCTTGTATGGGGATCATGAGTGCGG | 10 |
| | 1760 | 30 | GGAGCAGCGAGGGTAACATCTTGGCTCGGG | 16 |
| | 1809 | 16 | GGAAGAGGTTGGAGGG | 19 |
| E1 | 1981 | 30 | GGACGGAAAGATTCTCCATGAGATGGGCGG | 5 |
| | 2238 | 24 | GGTGGCCATATGAAACCAAGGTGG | 8 |
| | 2308 | 28 | GGCACTGAGGGATTTAACCAGGGTCTGG | 16 |
| E1-E2 | 2429 | 15 | GGGGCACAAGGCCGG | 16 |
| E2 | 2496 | 19 | GGCTACTCGGGGCCAGAGG | 15 |
| | 2565 | 28 | GGACCGTTAAGGCCATTTGCGTGGCAGG | 13 |
| | 2620 | 20 | GGTCAGTAGGAGGTATTTGG | 16 |
| | 2716 | 28 | GGAAATGGGAGATGACTTCGGGTTCGGG | 16 |
| | 3011 | 27 | GGTGAACCAGTGGTCTACACGGGGGGG | 15 |
| | 3475 | 21 | GGTGGTGGTAGCACTGTTAGG | 11 |
| E2-p7 | 3557 | 25 | GGTTTACCATTGGGCCAGGGTGAGG | 16 |
| p7-NS2 | 3748 | 24 | GGTTAGCGGGGTTGCCAGGGGTGG | 17 |
| NS2 | 3782 | 22 | GGCGGTTGGCAGCGGCTGCCGG | 20 |
| | 3892 | 29 | GGTTATAACGGTGGCAACCCTGAGAACGG | 9 |
| | 4069 | 26 | GGTACTGAAGGGAATAGGTGAGTTGG | 19 |
| | 4168 | 30 | GGTAACAAGATGGAATCTGGACATAGCCGG | 17 |
| | 4327 | 22 | GGCAGAAAGGGGCTGGTTATGG | 19 |
| | 4384 | 23 | GGTTGACCAAGCTGGTGAAGGGG | 10 |
| | 4743 | 25 | GGTTTGGTGACGAAGAGGTTTATGG | 15 |
| | 4979 | 26 | GGGCCGGTTAGAGAGGAGTACGCAGG | 16 |
| | 5097 | 12 | GGACGGAGGTGG | 20 |
| NS2-3 | 5116 | 27 | GGAACACCTTGGCTGGGTGCTTAGAGG | 16 |
| NS3 | 5289 | 20 | GGGGGTTAGAAACTGGCTGG | 13 |
| | 5371 | 28 | GGTATGTGACACTATGGGCCGGACAAGG | 11 |
| | 5462 | 21 | GGATGCCCGGAAGGAGCTAGG | 17 |
| | 5621 | 28 | GGCTGGTCAGGGCTACCGATATTTGAGG | 9 |
| | 5669 | 18 | GGCAGGGTCAAGGTCGGG | 20 |
| | 5825 | 30 | GGTGCCGGAAAAACCACGGAACTTCCTAGG | 16 |
| | 5992 | 21 | GGAAGGGGACATGGCCACAGG | 20 |
7

## Slide 8
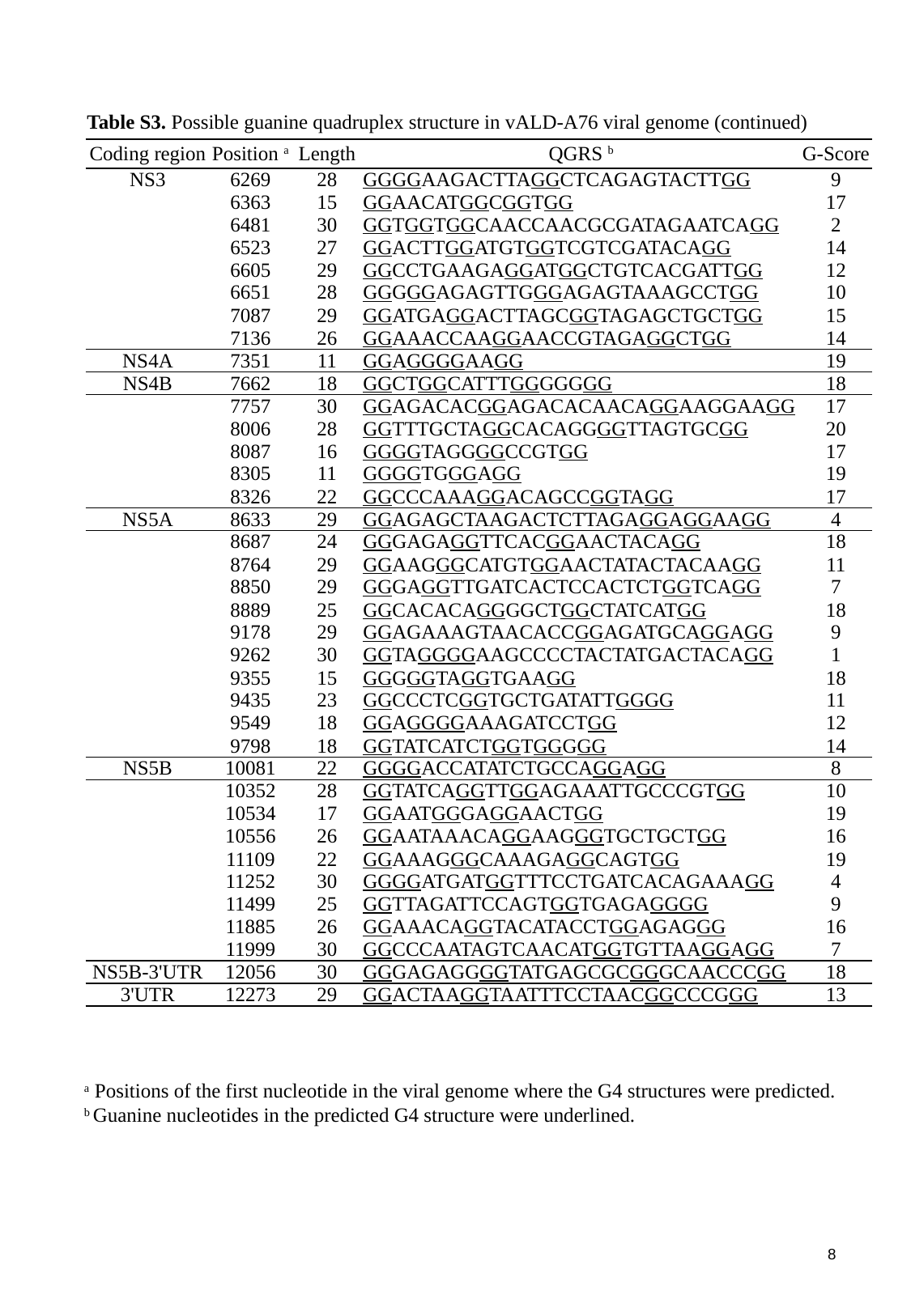

Table S3. Possible guanine quadruplex structure in vALD-A76 viral genome (continued)
| Coding region | Position a | Length | QGRS b | G-Score |
| --- | --- | --- | --- | --- |
| NS3 | 6269 | 28 | GGGGAAGACTTAGGCTCAGAGTACTTGG | 9 |
| | 6363 | 15 | GGAACATGGCGGTGG | 17 |
| | 6481 | 30 | GGTGGTGGCAACCAACGCGATAGAATCAGG | 2 |
| | 6523 | 27 | GGACTTGGATGTGGTCGTCGATACAGG | 14 |
| | 6605 | 29 | GGCCTGAAGAGGATGGCTGTCACGATTGG | 12 |
| | 6651 | 28 | GGGGGAGAGTTGGGAGAGTAAAGCCTGG | 10 |
| | 7087 | 29 | GGATGAGGACTTAGCGGTAGAGCTGCTGG | 15 |
| | 7136 | 26 | GGAAACCAAGGAACCGTAGAGGCTGG | 14 |
| NS4A | 7351 | 11 | GGAGGGGAAGG | 19 |
| NS4B | 7662 | 18 | GGCTGGCATTTGGGGGGG | 18 |
| | 7757 | 30 | GGAGACACGGAGACACAACAGGAAGGAAGG | 17 |
| | 8006 | 28 | GGTTTGCTAGGCACAGGGGTTAGTGCGG | 20 |
| | 8087 | 16 | GGGGTAGGGGCCGTGG | 17 |
| | 8305 | 11 | GGGGTGGGAGG | 19 |
| | 8326 | 22 | GGCCCAAAGGACAGCCGGTAGG | 17 |
| NS5A | 8633 | 29 | GGAGAGCTAAGACTCTTAGAGGAGGAAGG | 4 |
| | 8687 | 24 | GGGAGAGGTTCACGGAACTACAGG | 18 |
| | 8764 | 29 | GGAAGGGCATGTGGAACTATACTACAAGG | 11 |
| | 8850 | 29 | GGGAGGTTGATCACTCCACTCTGGTCAGG | 7 |
| | 8889 | 25 | GGCACACAGGGGCTGGCTATCATGG | 18 |
| | 9178 | 29 | GGAGAAAGTAACACCGGAGATGCAGGAGG | 9 |
| | 9262 | 30 | GGTAGGGGAAGCCCCTACTATGACTACAGG | 1 |
| | 9355 | 15 | GGGGGTAGGTGAAGG | 18 |
| | 9435 | 23 | GGCCCTCGGTGCTGATATTGGGG | 11 |
| | 9549 | 18 | GGAGGGGAAAGATCCTGG | 12 |
| | 9798 | 18 | GGTATCATCTGGTGGGGG | 14 |
| NS5B | 10081 | 22 | GGGGACCATATCTGCCAGGAGG | 8 |
| | 10352 | 28 | GGTATCAGGTTGGAGAAATTGCCCGTGG | 10 |
| | 10534 | 17 | GGAATGGGAGGAACTGG | 19 |
| | 10556 | 26 | GGAATAAACAGGAAGGGTGCTGCTGG | 16 |
| | 11109 | 22 | GGAAAGGGCAAAGAGGCAGTGG | 19 |
| | 11252 | 30 | GGGGATGATGGTTTCCTGATCACAGAAAGG | 4 |
| | 11499 | 25 | GGTTAGATTCCAGTGGTGAGAGGGG | 9 |
| | 11885 | 26 | GGAAACAGGTACATACCTGGAGAGGG | 16 |
| | 11999 | 30 | GGCCCAATAGTCAACATGGTGTTAAGGAGG | 7 |
| NS5B-3'UTR | 12056 | 30 | GGGAGAGGGGTATGAGCGCGGGCAACCCGG | 18 |
| 3'UTR | 12273 | 29 | GGACTAAGGTAATTTCCTAACGGCCCGGG | 13 |
a Positions of the first nucleotide in the viral genome where the G4 structures were predicted.
b Guanine nucleotides in the predicted G4 structure were underlined.
8
